# Aging-independent decrease of complex multi-spine boutons in hippocampal area CA1 after contextual fear conditioning

**DOI:** 10.1186/s13041-025-01265-z

**Published:** 2025-12-02

**Authors:** Raquel Martinez-Serra, Suji Lee, Igor Kraev, Karl Peter Giese

**Affiliations:** 1 Department of Basic and Clinical Neuroscience, King’s College London, London, UK; 2 Electron Microscopy Facility, Faculty of Science, Technology, Engineering and Mathematics, Open University, Milton Keynes, UK

**Keywords:** Multi-synapse bouton, Hippocampus, Memory, Aging

## Abstract

Long-lasting synaptic changes enable memory storage and regulate recall in the brain. Our previous work established that generation of multi-innervated dendritic spines (MISs), spines with typically two excitatory presynaptic inputs, underlies hippocampal memory formation in aged, but not young mice. The identification of MIS generation was done by ultrastructural analysis in hippocampal CA1 stratum radiatum 24 h after contextual fear conditioning (CFC). However, our analysis did not consider multi-spine boutons (MSBs), which were recently shown to increase in complexity (complex MSBs are pre-synaptic boutons connecting with more than two post-synapses) at a later time point after CFC in young age. Therefore, we re-analyzed our three-dimensional electron microscopy images and show that, unexpectedly, MSB complexity, decreases in CA1 stratum radiatum 24 h after CFC. The decrease in MSB complexity occurred both in young and aged mice, indicating that aging has no impact on this synaptic change. Considering that complex MSBs link the activity of multiple postsynaptic neurons, we suggest that after CFC a decrease in MSB complexity may be required for specific memory recall.

**Supplementary Information:**

The online version contains supplementary material available at 10.1186/s13041-025-01265-z.

## Introduction

The hippocampus has a critical role for memory [1], and with aging hippocampal memory formation declines [2]. However, hippocampal memory can still be formed and stored in old age. Using experimental conditions under which young and aged mice form equally a hippocampus-dependent contextual fear memory, we found evidence that the synaptic basis of memory changes with aging [3]. Specifically, we found that (structural) long-term potentiation (LTP) in hippocampal CA1 stratum radiatum underlies contextual memory formation in young mice, but not in aged mice. Instead, generation of multi-innervated spines (MISs), which involves attraction of a presynaptic input onto an existing dendritic spine [4], underlies contextual memory formation in aged, but not young mice. This finding not only illustrates that the synaptic basis of memory changes with age, it also points toward an importance of specific types of synaptogenesis that lead to generation of multi-synapses.

Most synapses exist in a 1–1 ratio between pre-synaptic and post-synaptic terminals. However, multi-synapses, connections between more than two synaptic profiles, have been observed in significant abundance in mammalian brains of several species, including humans [5]. The most abundant multi-synapses are multi-spine boutons (MSBs), where one pre-synaptic bouton connects to multiple spines. Our previous analysis [3] did not consider MSBs, even though MSB density is known to be increased after LTP induction in hippocampus [6–8] as well as after hippocampus-dependent trace eyeblink conditioning [9]. A very recent study assessed ultrastructural changes between synapses of engram and non-engram neurons and showed that the MSB complexity, the amount of post-synaptic spines per pre-synaptic bouton, increases after contextual fear memory formation in young mice [10]. This increase in MSB complexity was identified one week after contextual fear conditioning (CFC) and is in alignment with late changes in MSB complexity after hippocampal memory formation in a primate [11].

**Fig. 1:**
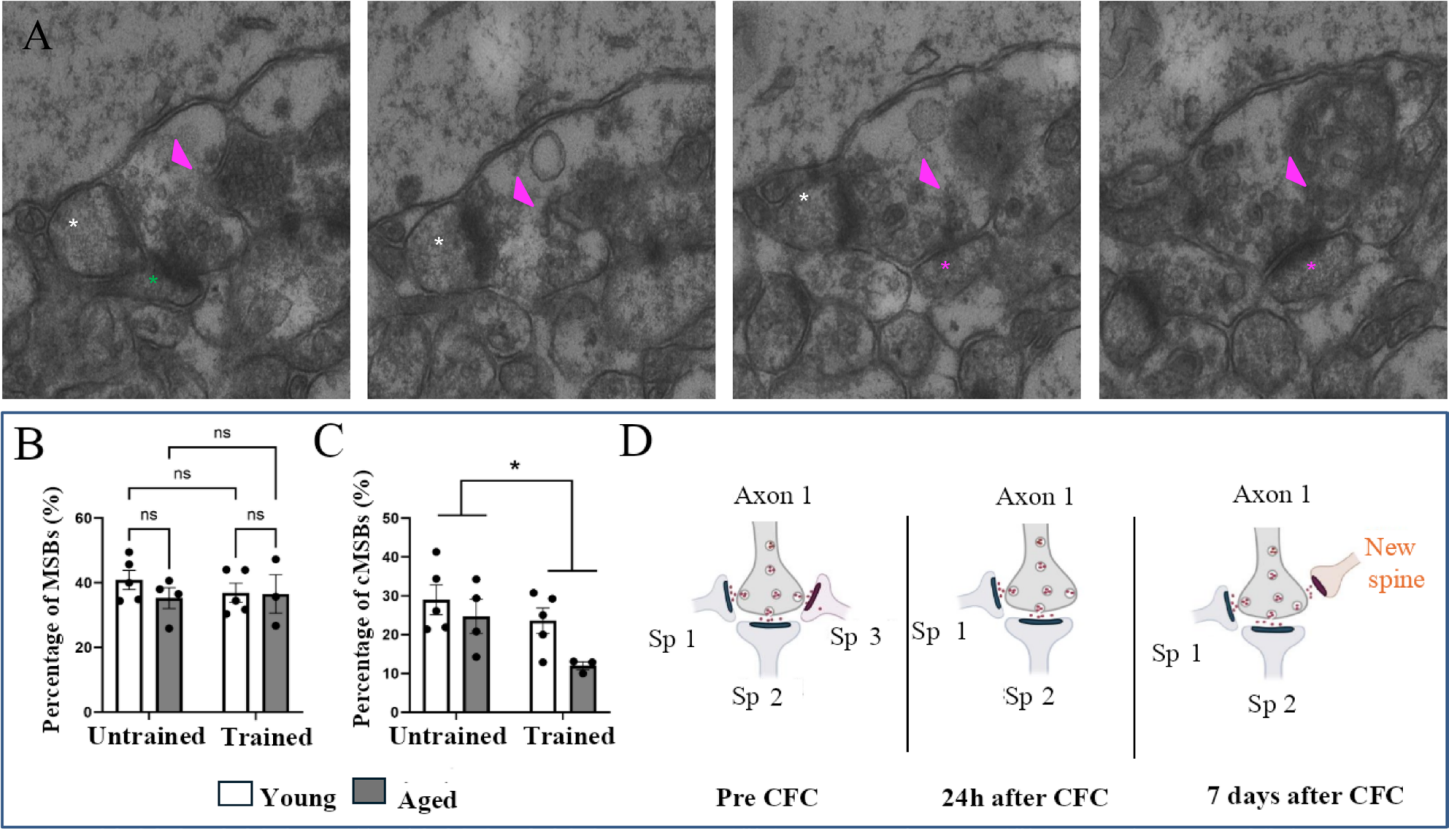
Decreased MSB complexity in CA1 stratum radiatum after contextual fear conditioning in young and aged mice. Young (3 month-old) and aged mice (18 month-old) underwent contextual fear conditioning and were killed 24 h after training for MSB analysis. MSB percentage and complexity were analysed in CA1 stratum radiatum. Effects of CFC training and ageing on multi-synaptic boutons were analysed. **A** Serial images showing a complex MSB with three spines indicated with * are connected to the same axonal bouton indicated with the arrowhead. **B** The MSB percentage did not change between the groups (two-way ANOVA; effect of ageing: F(1,13) = 0.67 *p* = 0.43; effect of training: F(1,13) = 0.14 *p* = 0.72; interaction age x training: F(1,13) = 0.52 *p* = 0.48). **C** Contextual fear conditioning reduced MSB complexity for both ages (two-way ANOVA; effect of ageing: F(1,13) = 4.4 *p* = 0.055; effect of training: F(1,13) = 5.8 *p* = 0.032; interaction age x training: F(1,13) = 0.93 *p* = 0.35). Data shown is mean ± S.E.M, *n* = 3,5 mice with 66–129 boutons analysed per mouse (Supplementary Table S1). **D** Schematic illustration of the proposed hypothesis with the number of spines (Sp) in complex MSBs decreasing 24 h after CFC, and increasing by incorporating non-engram cells (indicated in orange) 7 days after CFC

Here, we analyzed whether MSBs change in hippocampal CA1 stratum radiatum 24 h after CFC in young and aged mice, using previously obtained 3D electron microscopy images [3]. PSDs were already reconstructed before so for this study, just pre-synaptic boutons across synapses were reconstructed, with tracing stopping one section after pre-synaptic vesicles or PSDs were not visible. Pre-synapses were followed along serial images and classified as single-synaptic boutons (one pre-synapse connected to one post-synapse) or multi-synaptic boutons (one pre-synapse connected to many post-synapses) (Fig. 1A, supplementary Table 1). We found that MSB percentage (calculated as number of MSBs/total bouton number) did not change after CFC in both young and aged mice (Fig. 1B). The lack of an age-related decrease in MSB density in hippocampal area CA1 is different from the previously described age-related decrease in MSB density in dentate gyrus [11]. One potential reason for this difference is that hippocampal neurogenesis in dentate gyrus declines with ageing [12], and this could lead in general to a decrease in synaptogenesis in this hippocampal subregion. Moreover, our finding that MSB percentage does not change after CFC is consistent with a lack of change in MSB density at a later point after CFC [10]. Interestingly, these results are in contrast with an increase in MSB density after an LTP-inducing stimulation [6, 8]. As LTP is defined as strengthening of an existing synapse, an increase in MSB density is only an associated mechanism by the electrical stimulation. Further, the increase of MSB density after electrical stimulation was measured only up to 2 h, whereas the measurement of MSB density after CFC was measured at least after 24 h. Thus, it remains conceivable that an increase in MSB is part of transient synaptogenesis after CFC [13].

In contrast to no changes in MSB percentage, we found that MSB complexity was reduced in hippocampal area CA1 after CFC, both in young and aged mice (Fig. 1C). Considering that in young age there is no CFC-induced change in typical single-spine bouton (SSBs) and MIS density [3], the decreased MSB complexity indicates that removal of synaptic connections specifically from MSBs associates with contextual fear memory formation. Considering that mainly complex MSBs connect a pre-synaptic neuron with multiple post-synaptic neurons [14], a decrease in complex MSB density may allow for specific recall of newly established contextual fear memory (Fig. 1D). Consistent with this idea, at a later point when contextual fear memory becomes more gist-like [15], complex MSBs increase between engram and non-engram neurons [10]. Thus, our findings together with published work indicate that there is biphasic modulation of MSB complexity during contextual fear memory consolidation.

## Supplementary Information

Below is the link to the electronic supplementary material.

Supplementary Material 1.

Supplementary Material 2.

## Data Availability

No new data sets were generated in the current study.
