# Supplementary material for: Aging-independent decrease of complex multi-spine boutons in hippocampal area CA1 after contextual fear conditioning
Source: Mol Brain. 2025 Dec 2;19:1. doi: 10.1186/s13041-025-01265-z (PMC12777421; doi:10.1186/s13041-025-01265-z)
Supplement: Supplementary file 1 — Supplementary Material 1. [file 13041_2025_1265_MOESM1_ESM.docx]

**Supplementary Materials**

**Methods**

## **Three-dimensional electron microscopy for mouse samples**

Tissue preparation for EM was described in our earlier paper [1]. Briefly, 3-4 months old (young group) and 18 months old (aged group) female C57BL/6J inbred mice (Harlan, NL) were trained in contextual fear conditioning (CFC) with five mild foot shocks. The training schedule generated the same amount of contextual fear memory in young and aged mice. Twenty-four hours after CFC mice were culled by terminal anaesthesia and intracardially perfused with 3% PFA and 0.5% glutaraldehyde in 0.1 M phosphate buffer (pH 7.4) after the presence of contextual fear memory was confirmed. Brains were coronally cut into a 50 μm thick sections using a vibratome. Sections containing dorsal hippocampus were processed and were dehydrated in aqueous solutions of ethanol from 30% to 100%, for 10 minutes each, and then in 100% acetone, 3 times for 10 minutes each. Samples were then infiltrated with a combination of 50% pure acetone and 50% epoxy resin [Epon 812/Araldite CY212 epoxy resins] for 2 hours at RT. Slices were flat-embedded and polymerised for 48 hours at 60°C, then covered with a gelatine capsule and polymerised again for 48 hours at 60°C. Ultrathin serial sections (60–70 nm) were cut with a Diatome diamond knife and counterstained with 3.5% aqueous uranyl acetate followed by Reynolds’ lead citrate. Serial sections of 18 mm x 11 mm in the middle of the stratum radiatum region of the CA1 were imaged with a JEOL JEM1400 electron microscope using an AMT XR60 camera in montaging mode at a column magnification of 6000x.

## **Synaptic analysis for mouse samples**

Individual frames were merged using Photomerge command in Adobe Photoshop CS6, and to speed the process, a custom script was written using Adobe ExtendScript Toolkit. Next, merged images were aligned manually using SEM. Align 1.26b program (<http://synapseweb.clm.utexas.edu/>). Finally, dendritic spines and their PSDs were reconstructed using Reconstruct software (<http://synapseweb.clm.utexas.edu/software-0>). An unbiased brick method [2, 3] was used for the synapse analysis. The series were equally divided into 4 bricks of 16x8 μm, further divided into 20 sections each, with 10 sections gap between bricks. Three sides of each brick were considered inclusion planes, and the other three sides were exclusion planes. Synapses completely inside the brick or touching only inclusion planes were included in the analysis. To make counting easier, each brick was further divided into 2x2 μm squares for a total of 32 squares. Synapses were reconstructed in 2 out of these 32 squares which were selected using a random number generator.

PSDs were already reconstructed before [1]. Therefore, for this study, just pre-synaptic boutons across synapses were reconstructed, with tracing stopping one section after pre-synaptic vesicles or PSDs were not visible. Pre-synapses were followed along serial images and classified as single-synaptic boutons (one pre-synapse connected to one post-synapse) or multi-synaptic boutons (one pre-synapse connected to many post-synapses). MSBs were then further classified into simple MSBs (with just two post-synapses connected to one pre-synapse) and complex MSBs (with more than two post-synapses connected to the same pre-synaptic bouton). The criteria to determine whether two or more connections belonged to the same pre-synaptic bouton were also: visualisation of pre-synaptic vesicles, presence of PSD and distance between the two connections (1500 nm maximum). If an axonal bouton with just one connection could not be fully reconstructed, it was classified as uncategorised and excluded from the analysis, due to the impossibility to assess the presence of other connections. All analyses were done in a blinded manner and re-analysed by two independent researchers.

**Statistical analysis**

All data were analysed with GraphPad Prism 10.0.3. To assess for normality, Saphiro-Wilk test was used followed by a parametric or non-parametric test, depending on the results. The normal data was analysed using two-way analysis of variance (ANOVA) and Tukey’s multiple comparison. All data is presented as mean +/- standard error of the mean (SEM). Statistical significance thresholds were defined as: *p<0.05, **p< 0.01, ***p<0.001, ****p<0.0001.

**References**

1. Aziz W, Kraev I, Mizuno K, Kirby A, Fang T, Rupawala H, Kasbi K, Rothe S, Jozsa F, Rosenblum K, et al: **Multi-input synapses, but not LTP-strengthened synapses, correlate with hippocampal memory storage in aged mice.** *Curr Biol* 2019, **29**:3600-3610.
2. Fiala JC, Harris, KM: **Cylindrical diameters method for calibrating section thickness in serial electron microscopy.** *J Microsc* 2001, **202**:468–472.
3. Fiala JC, Harris, KM: **Extending unbiased stereology of brain ultrastructure to three-dimensional volumes.** *J Am Med Inform Assoc* 2001, **8**:1–16.
