# Supplementary figures and images for: Aging-independent decrease of complex multi-spine boutons in hippocampal area CA1 after contextual fear conditioning
Source: Mol Brain. 2025 Dec 2;19:1. doi: 10.1186/s13041-025-01265-z (PMC12777421; doi:10.1186/s13041-025-01265-z)

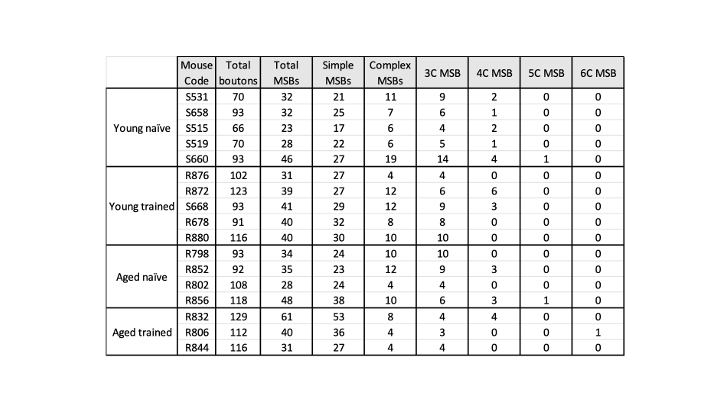

Supplement: Supplementary file 2 — Supplementary Material 2. [file 13041_2025_1265_MOESM2_ESM.tiff]
